# Human cytosolic transaminases: side activities and patterns of discrimination towards physiologically available alternative substrates

**DOI:** 10.1007/s00018-022-04439-3

**Published:** 2022-07-14

**Authors:** Francesco Caligiore, Erika Zangelmi, Carola Vetro, Takfarinas Kentache, Joseph P. Dewulf, Maria Veiga-da-Cunha, Emile Van Schaftingen, Guido Bommer, Alessio Peracchi

**Affiliations:** 1 De Duve Institute and WELBIO, UCLouvain, Avenue Hippocrate 75, 1200 Brussels, Belgium; 2 Department of Chemistry, Life Sciences and Environmental Sustainability, University of Parma, 43124 Parma, Italy

**Keywords:** Aminotransferases, Side reactions, Cytosolic malate dehydrogenase, Indolepyruvate, Enzyme compartmentalization

## Abstract

Transaminases play key roles in central metabolism, transferring the amino group from a donor substrate to an acceptor. These enzymes can often act, with low efficiency, on compounds different from the preferred substrates. To understand what might have shaped the substrate specificity of this class of enzymes, we examined the reactivity of six human cytosolic transaminases towards amino acids whose main degradative pathways do not include any transamination. We also tested whether sugars and sugar phosphates could serve as alternative amino group acceptors for these cytosolic enzymes. Each of the six aminotransferases reacted appreciably with at least three of the alternative amino acid substrates in vitro, albeit at usually feeble rates. Reactions with L-Thr, L-Arg, L-Lys and L-Asn were consistently very slow—a bias explained in part by the structural differences between these amino acids and the preferred substrates of the transaminases. On the other hand, L-His and L-Trp reacted more efficiently, particularly with GTK (glutamine transaminase K; also known as KYAT1). This points towards a role of GTK in the salvage of L-Trp (in cooperation with ω-amidase and possibly with the cytosolic malate dehydrogenase, MDH1, which efficiently reduced the product of L-Trp transamination). Finally, the transaminases were extremely ineffective at utilizing sugars and sugar derivatives, with the exception of the glycolytic intermediate dihydroxyacetone phosphate, which was slowly but appreciably transaminated by some of the enzymes to yield serinol phosphate. Evidence for the formation of this compound in a human cell line was also obtained. We discuss the biological and evolutionary implications of our results.

**Supplementary Information:**

The online version contains supplementary material available at 10.1007/s00018-022-04439-3.

## Introduction

Substrate specificity is a hallmark of enzyme catalysis; it consists in the ability to bind and transform one (or a few) preferred substrate(s) while discriminating against a variety of undesired compounds [1, 2]. Specificity is an evolved property: it is usually assumed that, during the earliest phases of life history, enzymes were broad-specificity catalysts capable of acting on many substrates. In the course of evolution, most of them tended to become more and more specific for a given metabolite [3–5]. A high selectivity towards a single substrate may offer several advantages, particularly for enzymes involved in central metabolic pathways: specialized enzymes are possibly more efficient, and they permit a finer regulation of metabolism and prevent interferences between different pathways [6].

Nonetheless, it has long been known that a perfect substrate specificity is essentially unattainable, as there are chemical–physical limits to the maximum achievable discrimination between similar substrates [7, 8]. Furthermore, such limits are seldom approached by extant enzymes, due in all likelihood to evolutionary factors (such as an insufficient selective pressure) [9]. As a consequence, the enzymes of today’s organisms retain often an appreciable capacity to transform alternative substrates that show some chemical and structural similarity with the physiological (preferred) ones [9–11].

Sometimes, the minor reactions of abundant enzymes with alternative substrates may contribute to secondary (but established) metabolic pathways that have some importance in the organism under examination. In humans, an example exists in the biosynthesis of carnitine. One of the steps required for this process is the formation of 4-*N*-trimethylaminobutyraldehyde from hydroxyl-trimethyllysine, through an aldolase reaction. However, no dedicated aldolase has ever been isolated and it is believed that, in humans, the reaction is catalyzed as a side job by serine hydroxymethyltransferase [12]. More often, the reactions with alternative substrates can lead to unusual products that have no known usefulness or are even potentially harmful. In some instances, such detrimental products can be recycled or destroyed by dedicated ‘repair’ enzymes (e.g., Refs. [13–15]), while in other instances, they may be excreted as such, or undergo further transformations before being excreted.

Minor reactions may come to the forefront under certain pathological circumstances, especially in the presence of inherited metabolic defects. For example, alternative reactions may be favored by mutations that decrease the specificity of an enzyme (as in hereditary sensory autonomic neuropathy type 1 [16, 17]) or when the preferred substrate of the enzyme is depleted (as in the hyperornithinemia–hyperammoniemia–homocitrullinuria syndrome [18]). Also, the products of side reactions can build up when the concentration of the alternative substrate is abnormally high (the classic example being phenylketonuria [19, 20]) or when such products are not removed due to the deficiency of a dedicated repair enzyme (such as in L-2-hydroxyglutaric aciduria [21, 22]).

Here, we explore the side activities of human cytosolic aminotransferases (transaminases). These enzymes catalyze the transfer of the amino group from a donor (most commonly, an α-amino acid) to an acceptor that bears a carbonyl function. The human genome contains genes for some 15 different transaminases, at least six of which are localized predominantly or solely in the cytosol [23]. As transaminases are notoriously prone to react with alternative substrates, we focused on the capacity of the cytosolic aminotransferases to act on a series of alternative amino group donors and amino group acceptors normally available in the cytosol.

It was anticipated that this research could be interesting under different viewpoints. First, it should provide a better understanding of the actual degree of specificity to which these transaminases have been shaped by evolution, both as single enzymes and as a group, providing hints as to which side activities have been more suppressed during evolution. Furthermore, it may allow an estimate of the efficiency of the transamination reactions with ‘non-preferred’ substrates in vivo, and of the relative contribution of different transaminases to such reactions. Finally, this study may also suggest the plausible origin of some compounds that have been detected (and of some others that could be detected in the future) in the human metabolome. This may be of particular interest for compounds related to pathological phenotypes.

## Materials and methods

### Commercial enzymes and chemicals

Bovine liver glutamate dehydrogenase (GDH) was purchased from Sigma-Aldrich (now Merck, Darmstadt, Germany) or Roche (Mannheim, Germany). Pig heart malate dehydrogenase was from Roche. Indolepyruvic acid (IndPy) was from Carbosynth (Staad, Switzerland). TLC plates (silica gel 60 F254) were from Merck Millipore. Solvents for LC–MS were from Biosolve BV, The Netherlands. All other reagents were from Fluka or Sigma-Aldrich (now Merck, Darmstadt, Germany).

### Recombinant enzymes

The cloning and expression of the human cytosolic transaminases used in this work (listed in Supplementary Table 1) has been described previously [24]. For the present study, the clone of the human tyrosine aminotransferase (TAT) was modified by removing the first 40 codons of the coding sequence, to obtain a shortened recombinant protein similar to that which yielded the crystal structure of TAT (PDB: 3DYD). The removed tail of amino acids is possibly important for the ubiquitin-mediated regulation of TAT in vivo*,* as it is identified as TAT_ubiq in the Pfam database [25]; however, its ablation does not impair activity [26] and improves substantially the yield of soluble recombinant protein.

The clone of human lactate dehydrogenase A (LDHA) was a kind gift from Dr. Lisa Craig (Simon Fraser University, Burnaby, Canada) [27]. The clone of human cytosolic malate dehydrogenase (MDH1; NCBI accession number NP_005908) was obtained in the present study; the protein coding sequence was PCR-amplified from human kidney cDNA and ligated into a pET28a expression vector. After sequence verification, the recombinant enzyme was expressed in *E. coli* BL21(DE) cells. Recombinant yeast ω-amidase (product of the yNit3 gene) was produced as described [14].

All the recombinant His6-tagged proteins were purified by metal-affinity chromatography [20, 23]. The enzymes were then transferred to an appropriate storage buffer (typically 50 mM Hepes pH 7.5, 100 mM NaCl, 0.5 mM EDTA, 1 mM DTT, 10% glycerol; in the case of aminotransferases, the buffer was also supplemented with 5 μM pyridoxal 5’-phosphate—PLP) and kept frozen at − 80 °C.

### Preliminary assessment of the reactions of cytosolic transaminases with alternative amino group donors (TLC assay)

The recombinantly expressed transaminases were preliminarily tested for reactivity towards alternative amino acid substrates (L-Lys, L-Arg, L-Thr, L-Asn, L-His and L-Trp; Supplementary Tables 2 and 3) using some common amino group acceptors (typically, α-ketoglutarate, pyruvate and glyoxylate). This quick semi-quantitative assay exploited thin-layer chromatography (TLC) and relayed on the experimental observation that five of the amino acid substrates (with the exception of L-Thr) could be clearly separated from the expected products (L-Glu, L-Ala or Gly) on TLC plates. On the other hand, since L-Thr migrated too close to either L-Glu, L-Ala or Gly, phenylpyruvate (whose transamination would generate L-Phe) was used as an acceptor in the initial L-Thr transamination assays.

Reactions with a final volume of 200 μL were carried out in 0.5 mL Eppendorf tubes. The reaction buffer was 20 mM Na-phosphate (pH 8.0), also containing 50 mM NaCl and 0.5 mM DTT. The amino acid concentration was 5 mM, the concentration of the amino group acceptor was also 5 mM, and the concentration of enzyme was 5 μM. The reaction tubes were incubated for ~ 16 h at 37 °C, together with controls in which the enzyme had been omitted, to exclude the occurrence of non-enzymatic reactions.

At the end of the incubation, 1 µL of the reaction mixture was spotted on a thin-layer silica gel plate and developed with acetic acid:1-propanol:distilled water—1:3:1. After the chromatographic separation, amino group-containing compounds were visualized by spraying the plates with 0.3% ninhydrin in methanol, drying, and heating for about 5 min. The plates were then photographed. Each experiment was performed in triplicate. An example of TLC assay is provided in Supplementary Fig. 1. Control experiments indicated that this assay could detect the formation of amino acid products at concentrations as low as 0.2 mM (or 4% of the initial substrate concentration).

### Separation of the keto acid products by LC–MS

In several instances, formation of the correct transamination products for the six amino acids (L-Lys, L-Arg, L-Thr, L-Asn, L-His and L-Trp) was confirmed through LC–MS analysis (Supplementary Figs. 2 and 3).

For these analyses, each enzyme–substrate couple was incubated overnight in a solution containing 20 mM Hepes buffer (pH 8.0) and 0.5 mM DTT. The concentration of the amino acid was 4 mM, while the concentration of keto acid was typically 1 mM. The tubes containing these reaction mixtures were equilibrated at 37 °C before starting the reaction by adding the enzyme (up to 5 μM final concentration; total reaction volume: 200 μL). Aliquots of the reaction mixture (5 μL each) were collected at appropriate intervals and diluted 80-fold by mixing with 395 μL MilliQ water. Immediately thereafter, the diluted aliquots were heated at 100 °C for 5 min to completely inactivate and precipitate the aminotransferase. After centrifuging for 10 min at 10,000 × *g* to remove the precipitated protein, 50 μL of the supernatant were recovered and loaded in vials for LC–MS injection on a 1290 HPLC system coupled to an ESI-QTOF iFunnel 6550 series MS (Agilent technologies).

LC–MS separation of the transamination products for basic amino acids (L-Lys, L-Arg and L-His) was performed on a Kinetex F5 2.6 µm 2.1 mm × 150 mm column (Phenomenex, Maarsen, The Netherlands), through a procedure loosely based on the protocol described by Bonte and coworkers [28]. For L-Asn, L-Thr and L-Trp, the chromatographic separation of the transamination products was performed on an ODS-4 column (150 × , 2.1 mm; GL Biosciences). Details of the LC–MS procedures are provided in the Supplementary Methods.

### Kinetic assays

To assess the actual rates of each transamination reaction, individual assays had to be developed, as detailed below. For all assays involving the human transaminases except GTK, α-KG was typically used as the amino group acceptor. For GTK, which reacts extremely poorly with α-KG, pyruvate was used as the amino group acceptor. Pyruvate was preferred to 2-keto-4-methylthiobutyrate (KetoMet) or phenylpyruvate, which are the best substrates (Supplementary Table 2) but are not very soluble and give substrate inhibition at modest concentrations [29]. α-KGM (the ketoacid corresponding to L-Gln) also could not be used, because it tends to cyclize in an unreactive form. To compensate in part for the relatively modest efficiency of pyruvate as a substrate of GTK, the keto acid was used at a concentration of 3 mM, which is ~ 15-fold higher than the concentration in hepatocytes (Supplementary Table 3).

Kinetic data were analyzed by nonlinear least-squares fitting to the appropriate kinetic equation (e.g., the Michaelis–Menten equation) using Sigma Plot (Systat Software Inc.).

### Threonine transamination assay

L-Thr transaminase reactions were studied spectrophotometrically, exploiting the fact that the reaction product, 3-hydroxy-2-ketobutyrate, is reduced by LDHA (albeit inefficiently) with the concomitant oxidation of NADH (see “Results” section and Supplementary Table 4). However, the poor efficiency of LDHA in this reaction, combined with the fact that the L-Thr transamination reactions were also consistently very slow, made it impractical to use LDHA in a continuous coupled assay. Thus, formation of 3-hydroxy-2-ketobutyrate was quantitated using LDHA in a discontinuous (end-point) assay, the details of which are provided in the Supplementary Methods.

### Lysine transamination assay

The rate of the transamination of L-Lys (the expected reaction is depicted in Supplementary Table 2) was measured by a discontinuous assay where substrates and products were quantitated by LC–MS. The choice of this method obliged us to limit the concentrations of salts and substrates in the assay mixture.

Reactions were performed in a solution containing 20 mM Hepes buffer (pH 8.0) and 0.5 mM DTT. The concentration of L-Lys ranged between 0.5 and 4 mM, while the concentration of keto acid was 0.5 mM (α-KG) or 3 mM (pyruvate). The reaction mixtures were equilibrated at 37 °C before starting the reaction by adding the enzyme (typically at 5 μM final concentration; total reaction volume: 200 μL). Aliquots of the reaction mixture (5 μL each) were collected at appropriate intervals, diluted 80-fold in MilliQ water and heated at 100 °C for 5 min to completely inactivate and precipitate the aminotransferase. After centrifuging for 10 min at 10,000 × *g*, 50 μL of the supernatant were recovered and used for the LC–MS analysis.

Separation of the analytes was performed on a Kinetex F5 column with the MS operated in positive mode, as described above. Substrates and products were quantitated based on the peak areas of extracted ion chromatograms showing the expected *m/z* of the [M-H^+^] ion. As different analytes generate different signals, the correlation between peak area and analyte concentration was established for L-Lys and L-Glu by comparison with a series of standards at known concentrations. The peak areas increased linearly with amino acid concentration and proportionality constants (slopes) were derived from the least-squares linear regression of the data. For piperideine-2-carboxylate (the cyclized form of the L-Lys keto acid; Supplementary Table 2), the correlation was established by comparing the area of its peak with that of the L-Glu peak, generated (in equimolar amounts) during the same transamination reactions. Ultimately, these comparisons established that the area/concentration proportionality is 4.5-fold higher for piperideine-2-carboxylate than for parent L-Lys. In other words, in a single time-point obtained at time t, the product-to-substrate concentration ratio can be estimated based on their peak areas according to the equation

1 $$\frac{{ \left[ P \right]_{t} }}{{ \left[ S \right]_{t} }} = \frac{{PA_{t} }}{{Q \times SA_{ t} }};\qquad \left( {Q = 4.5} \right)$$

where *PA*_*t*_ is the area of the product (piperideine-2-carboxylate) peak, *SA*_*t*_ is the area of the substrate (L-Lys) peak, and *Q* is the 4.5-fold normalization factor mentioned above.

Accordingly, the actual concentration of product at time *t*, [*P*]_*t*_, was calculated as follows:

2 $$\left[ P \right]_{t} = \left[ S \right]_{0} \frac{{PA_{ t} }}{{PA_{t} + \left( {Q \times SA_{ t} } \right)}}$$

where [*S*]_0_ is the initial concentration of the substrate (at *t* = 0). Of note, this calculation assumes that the amount of product existing in linear form (see Supplementary Table 2) is negligible with respect to the cyclic form [30], and that the keto acid product does not undergo any further transformation in the reaction mixture.

### Arginine transamination assay

The rate of the transamination of L-Arg was measured by a discontinuous assay essentially identical to that described above for L-Lys. In the reaction mixture (20 mM Hepes buffer—pH 8.0, 0.5 mM DTT), the concentration of L-Arg ranged between 0.5 and 4 mM, and the concentration of keto acid was 0.5 mM (α-KG) or 3 mM (pyruvate). The reactions were conducted as described above. Analyses of the chromatogram indicated that the peak area/concentration proportionality was 1.43-fold higher for the product 2-keto-5-guanidino valerate (also called ketoarginine and abbreviated here as KetoArg; Supplementary Table 2) [31] compared to the substrate L-Arg. Accordingly, the amount of product at any given time was estimated from Eq. 2 (using *Q* = 1.43).

### Asparagine transamination assay

L-Asn transaminase reactions, which generate α-ketosuccinamate (α-KSM), were monitored through a continuous coupled assay in which ω-amidase and MDH1 (the human cytosolic malate dehydrogenase) were the coupling enzymes. MDH1 itself showed no appreciable activity towards α-KSM. However ω-amidase converted α-KSM to oxaloacetate, which could then be reduced to L-malate by MDH, with the concomitant oxidation of NADH. Experimental details are provided in the Supplementary Methods.

### Tryptophan transamination assay

Based on the unexpected observation that IndPy (the keto acid deriving from L-Trp transamination) is reduced with good efficiency by the human MDH1 (see “Results” section and Supplementary Fig. 4), the reactions of the six transaminases with L-Trp were monitored through a continuous coupled assay with this enzyme. Details are provided in the Supplementary Methods.

### Histidine transamination assay

The reaction of L-His with the six aminotransferases tested was measured through a continuous assay that monitored the accumulation of the keto acid product, imidazolepyruvate (ImPy). ImPy absorbs at 286 nm, and using the pure compound, we determined an ε_286_ of 1400 M^−1^ cm^−1^ (Supplementary Fig. 5). Based on this, we could directly relate the increase in absorbance at 286 nm to the increase in concentration of the keto acid. The assay was performed in quartz cuvettes, equilibrated at 37 °C in a thermostatted spectrophotometer. The reaction buffer was 50 mM sodium phosphate (pH 8.0) also containing 50 mM NaCl. L-His concentrations ranged typically between 1 and 10 mM, whereas the concentration of the amino group acceptor was 0.5 mM (α-KG) or 3 mM (pyruvate). The concentration of the transaminase was 0.2–2.5 μM (depending on the enzyme under assay).

### Assay for the GTK-catalyzed transamination of different keto acids

The transamination reactions of GTK with different keto acids, using L-Gln as the amino group donor, were monitored through a coupled assay with ω-amidase and GDH. In this assay, transamination of L-Gln generates α-ketoglutaramate (α-KGM); ω-amidase hydrolyzes this compound to α-KG, which can be then converted to L-Glu by GDH. Experimental details are provided in the Supplementary Methods.

### Testing the possible reactions of the transaminases with sugars and sugar phosphates (GDH/LDHA assays)

The recombinant human transaminases were also preliminarily tested for reactivity towards alternative amino group acceptors (such as DHAP, GAP, etc.) using a discontinuous assay that detects the formation of keto acid products (α-KG or pyruvate).

Reactions (final volume of 640 μL) were carried out in 1.5 mL Eppendorf tubes. The reaction buffer was 20 mM Hepes buffer (pH 7.2), also containing 0.5 mM DTT. The amino group acceptor concentration was 5 mM; the amino group donor was either L-Glu (typically 1 mM) or L-Ala (3 mM); and the concentration of enzyme was 1–5 μM. The reaction tubes were incubated for 16 h at 37 °C, together with controls in which the enzyme was omitted.

At the end of the incubation, 600 µL of the reaction mixture were mixed in a plastic cuvette with 200 µL of a solution containing potassium phosphate pH 8.0 (50 mM final concentration), NADH (~ 0.19 mM final concentration), and ammonium chloride (10 mM final). After equilibrating the mixture for a few minutes at 30 °C, GDH (1U) was added and the overall decrease in absorbance at 340 nm could be related to the amount of α-KG formed during the 16-h incubation. We estimate that the assay could reliably detect the formation of keto acid products at concentrations as low as 16 μM (corresponding to 0.3% of the initial amino group acceptor concentration).

### Separation of serinol phosphate by LC–MS

The actual production of serinol phosphate, the expected product of DHAP transamination, was supported by LC–MS analysis. In these experiments, PSAT1 (5 μM) was incubated for 48 h with DHAP (5 mM) and L-Glu (2 mM) in 20 mM Hepes pH 7.2–1 mM DTT, at 37 °C. At the end of incubation, 20 μL of the reaction mixture were taken, and further reaction was quenched by adding (serially) 80 μL cold water, 100 μL dry-ice-cold methanol and 200 μL dry-ice-cold chloroform. After three vortexing cycles, phase separation was achieved by centrifuging at 25,000 *g* for 10 min at 4 °C. The (upper) aqueous phase was recovered and used to perform LC–MS analysis as described above using the ODS-4 column.

### Preparation of HEK293T cell extracts for LC–MS analysis

HEK293T cells were seeded at 350,000 cells/well in 6-well plates and grown for 48 h, with one medium change 24 h before analysis. The culture medium was DMEM 4.5 g/L Glucose from Lonza (Walkersville, MD, USA; according to the manufacturer, this medium contained 19.455 μM pyridoxine), supplemented with Fetal Bovine Serum (10% final) and UltraGlutamine™ (Lonza; 2 mM final).

For analysis, after one rapid wash with ice-cold water, culture plates were submerged in liquid nitrogen. Subsequently, 250 μL of a solution consisting of 90% methanol (Biosolve) and 10% chloroform was added, and lysates were transferred into microcentrifuge tubes. After centrifugation for 15 min at 4 °C and 22,000 *g*, the supernatant was recovered, dried in a SpeedVac vacuum concentrating system (Life technologies) and resuspended in 40 μL of water before analysis by LC–MS as described above.

## Results

In this work, we explored the reactivity towards alternative substrates of six human cytosolic transaminases: GOT1, GPT, GTK (product of the human gene KYAT1), TAT, PSAT1 and BCAT1 [20]. Relevant information about these enzymes, including the reactions they normally catalyze and their relative abundance in the cell, is given in Supplementary Table 1. These six enzymes represent almost the complete set of human cytosolic aminotransferases. A previous study had identified nine candidate cytosolic transaminases in the human genome [20], but it was subsequently shown that two of these enzymes (AGXT2L1 and AGXT2L2) were not transaminases, but rather lyases acting on phosphoethanolamine and phosphohydroxylysine, respectively [32]. The function of another cytosolic enzyme (GOT1L1) is debated [33–35], but in any case, neither us [20] nor others [34] were able to obtain a significant amount of soluble His-tagged GOT1L1 from bacteria, precluding its use in this study. Finally, yet another transaminase, GTL (glutamine transaminase L; product of the human gene KYAT3), is annotated in the Uniprot database [36] as localizing in the cytosol, as well as in the mitochondria (such a dual location is similar to that of GTK [37] and presumably depends on the alternative splicing of the initial gene transcript). Although we did not test the human GTL/KYAT3 in this study, a few control experiments were conducted with the mouse enzyme.

### Preliminary test of the reactivity of transaminases towards alternative amino acid substrates

We were interested in the ability of the cytosolic transaminases to act on amino acid substrates different from the preferred ones. We investigated L-Lys, L-Arg, L-Thr, L-Asn, L-Trp and L-His, whose main degradation pathways in human cells do not include transamination. The products expected from their transamination are shown in Supplementary Table 2, whereas the concentrations of these amino acids in liver and skeletal muscle are given in Supplementary Table 3.

**Fig. 1:**
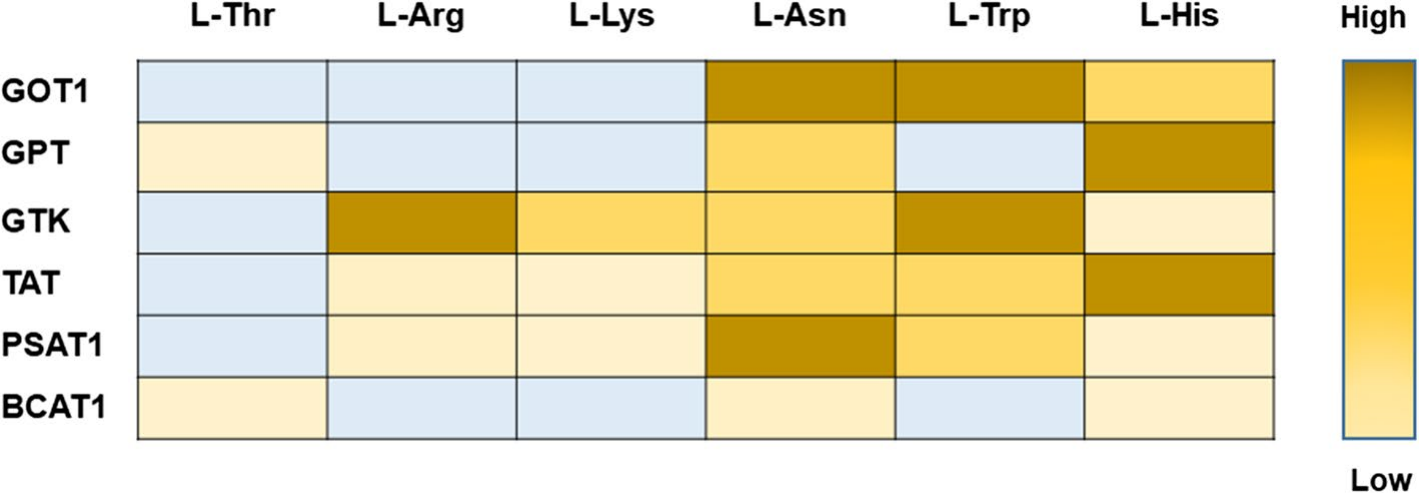
Cytosolic transaminases can act appreciably on amino acids whose main degradation pathways do not include transamination. This initial assessment is based on TLC assays described in the Materials and methods. Each enzyme (5 μM) was incubated at 37 °C with the specified amino acid substrate (5 mM) and with an amino group acceptor, and after ~ 16 h product formation was assessed by TLC. The extent of the transamination for each enzyme/amino acid combination is indicated semi-quantitatively by the intensity of the color. Light blue cells correspond to cases in which activity was not detected (given the sensitivity of the assay, this means that < 0.2 mM of the alternative substrate was transaminated in 16 h)

We initially used thin-layer chromatography (TLC) to test the reactivity of the six transaminases on the alternative substrates, obtaining a general, qualitative picture of their efficiency in catalyzing non-canonical transaminations. The results are summarized in Fig. 1.

This picture is very preliminary, since it relies on a single readout taken after a very long incubation—during which, for example, L-Asn might undergo some spontaneous deamination to form L-Asp. It also ignores factors such as substrate and product inhibition, as well as progressive inactivation of the enzymes, which might have contributed to the final amount of product formed in each case. Even with these caveats, the results suggested that all six amino acids tested could react with at least two cytosolic aminotransferases and that, conversely, each aminotransferase tested could react appreciably with at least three of the alternative (‘non-preferred’) amino acid substrates.

### Testing the possible activity of two human cytosolic dehydrogenases with the transamination products: MDH1 efficiently reduces indolepyruvate (IndPy)

To better understand the activities of those aminotransferases that reacted appreciably with the alternative substrates, we tested whether two human α-hydroxy acid dehydrogenases (which we had also produced in recombinant form) could reduce the expected products of the side transamination reactions. The dehydrogenases were lactate dehydrogenase A (LDHA) and the cytosolic malate dehydrogenase (MDH1). Our tests had two purposes. First, on the practical side, developing coupled spectrophotometric assays based on these dehydrogenases could facilitate the kinetic analysis of the reactions carried out by the transaminases with the alternative substrates. Second, evidence of activity of these dehydrogenases towards the transamination products could also have some relevance in vivo, since LDHA and MDH1 share with the six aminotransferases the same cytosolic localization.

In particular, preliminary assays established that the product of L-Thr transamination, 3-hydroxy-2-ketobutyrate, is a substrate for LDHA, which (consistent with a much earlier report on a rabbit enzyme [38]) is a rather promiscuous enzyme, as summarized in Supplementary Table 4. However, the modest efficiency of LDHA in the reduction of 3-hydroxy-2-ketobutyrate, combined with the fact that the L-Thr transamination reactions were also consistently very slow, made it impractical to use LDHA in a continuous coupled assay. Thus, formation of the keto acid in L-Thr transamination reactions was ultimately assessed employing LDHA in a discontinuous assay (see “Materials and methods” section).

During our tests, we made the unexpected observation that recombinant MDH1 reduced with good efficiency IndPy (the keto acid deriving from L-Trp transamination). The apparent *k*_cat_*/K*_M_ for reduction of this substrate approached 10^4^ M^−1^ s^−1^, which is substantial in absolute terms despite being 250-fold lower than *k*_cat_*/K*_M_ for the preferred substrate oxaloacetate (Supplementary Fig. 4A–C). IndPy was *not* reduced by the most common preparation of commercial MDH, from pig heart mitochondria (data not shown; note that the sequence identity between cytosolic and mitochondrial MDH enzymes is < 26%). The observed reduction of IndPy was inhibited by compounds structurally similar to oxaloacetate, arguing against the contamination of our recombinant MDH1 preparation by some other dehydrogenase (Supplementary Fig. 4D–E). Based on these observations, MDH1 was used to monitor the L-Trp transamination reactions.

### Efficiency of the reactions of the transaminases with alternative amino acid substrates

**Fig. 2:**
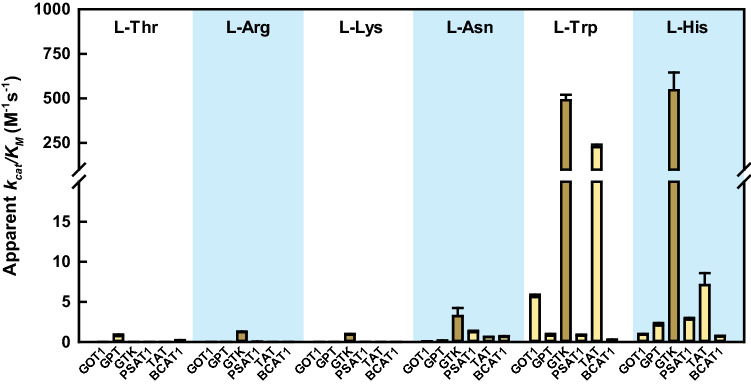
Apparent reaction efficiency (*k*_cat_*/K*_M_) for the transaminations catalyzed by the six cytosolic transaminases with alternative amino acid substrates. Activities were tested at pH 8.0 as described in the Materials and methods, using as a co-substrate either α-KG (0.5 mM, yellow bars) or, for GTK only, pyruvate (3 mM, brown bars). *k*_cat_*/K*_M_ parameters were obtained as described in the legend of Supplementary Table 5 and are deemed ‘apparent’, because they were measured in the presence of a fixed concentration of co-substrate. Error bars represent the standard error of the fitting. For those reactions that were measured repeatedly on different days, the calculated kinetic parameters differed by less than 25% between individual measurements. Note that the reaction efficiencies of the transaminases towards the ‘physiological’ substrates are in general around 10^5^ M^−1^ s^−1^. For example, GOT1 (with the amino group donor L-Asp and the acceptor α-KG) shows a *k*_cat_*/K*_M_ = 0.63 × 10^5^ M^−1^ s^−1^ [39]; for GPT (substrates L-Ala and α-KG) *k*_cat_*/K*_M_ = 0.7 × 10^5^ M^−1^ s^−1^ [39]; for TAT (substrates L-Tyr and α-KG) *k*_cat_*/K*_M_ = 1.6 × 10^5^ M^−1^ s^−1^ [26]

Figure 2 shows the apparent reaction efficiency (*k*_cat_*/K*_M_) for many of the enzyme/substrate couples (the actual numerical values are found in Supplementary Table 5). Apparent *k*_cat_*/K*_M_ parameters were obtained by fitting the data (activity *vs.* substrate concentration) to the Michaelis–Menten equation. For many enzyme–substrate couples, saturation was not approached even at the highest amino acid concentration tested, preventing determination of accurate individual values for *k*_cat_ and *K*_M_. However, even in these cases, the *k*_cat_*/K*_M_ ratio could be reliably estimated, as it represents the slope of the initial part of the Michaelis–Menten curve (see legend of Supplementary Table 5).

### Activity of GTK towards the keto acids of L-His and L-Trp

Whereas the vast majority of activities summarized in Fig. 2 and Supplementary Table 5 were very weak (apparent *k*_cat_*/K*_M_ values < 10 M^−1^ s^−1^), three cases stood clearly out. One involved the rather efficient reaction of GTK with L-His; the other two involved the efficient transaminations of L-Trp by GTK and TAT.

**Fig. 3:**
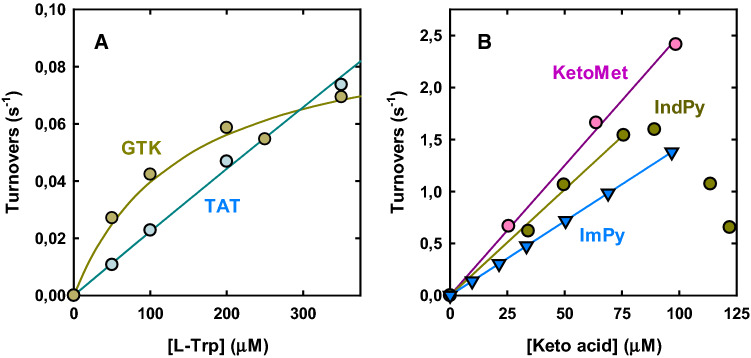
The efficiencies of GTK in transaminating L-Trp and the corresponding ketoacid, IndPy, suggest a role of the enzyme in L-Trp salvaging. **A** Transamination of L-Trp catalyzed by GTK and TAT. The amino group acceptor was pyruvate (3 mM) in the case of GTK and α-KG (0.5 mM) in the case of TAT. The catalytic parameters extracted from fitting of the data are reported in Supplementary Table 5; in particular, apparent *k*_cat_*/k*_M_ values were 490 M^−1^ s^−1^ (GTK) and 226 M^−1^ s^−1^ (TAT). **B** Comparison of the activity of GTK towards different keto acids, including the physiological substrate KetoMet (in vivo, transamination of this amino acid is particularly important for closing the methionine salvage pathway [42]). The amino group donor was L-Gln (1 mM) in all cases. The decrease in activity of the enzyme at high IndPy concentrations is due to substrate inhibition. The *k*_cat_*/K*_M_ values obtained from the linear fitting of the data (solid lines) were 20,400 M^−1^ s^−1^ (IndPy) 14,300 M^−1^ s^−1^ (ImPy) and 25,000 M^−1^ s^−1^ (KetoMet). For TAT, too, the transamination of IndPy was one order of magnitude faster than the transamination of L-Trp

The reaction of a mammalian TAT with L-Trp had been reported before [40] and the present results support the possibility that the enzyme could be involved in some minor pathway for L-Trp degradation (see “Discussion” section). At first sight, the comparable *k*_cat_*/K*_M_ shown by GTK might suggest that this enzyme, too, plays some role in the degradation of L-Trp. However, a closer examination of the data revealed divergent kinetic features for the two enzymes. While GTK shows a rather low apparent *K*_M_ and low apparent *k*_cat_, the opposite is true for TAT (Fig. 3A). Thus, the synthetic parameter *k*_cat_*/K*_M_ in this case fails to adequately represent the very distinct reactivity of GTK and TAT towards L-Trp [41].

The peculiar kinetic behavior of GTK seems to imply a distinct metabolic function. Indeed, the main established role of GTK is the regeneration of amino acids, in particular L-Phe and L-Met, from the respective keto acids, at the expense of L-Gln [42], so we wondered whether the enzyme could also regenerate efficiently L-His and L-Trp. We therefore tested the reaction of GTK with the keto acids imidazolepyruvate (ImPy) and indolepyruvate (IndPy) (Fig. 3B). The apparent *k*_cat_*/K*_M_ with which GTK reconverted IndPy into L-Trp was about 40-fold higher than the efficiency of L-Trp transamination. Of note, while the IndPy transamination in Fig. 3B is not exactly the opposite of the reaction in Fig. 3A (because the co-substrate changes), its high efficiency seems biologically relevant, because the concentration of the co-substrate L-Gln in vivo is substantially higher than the concentration used in our assays (Supplementary Table 3). Interestingly, the efficiency of IndPy transamination also appeared to approach the efficiency shown by GTK towards KetoMet (Fig. 3B), which is generally regarded as a preferred substrate of this enzyme [42].

### Testing the reactivity of the transaminases towards sugars and sugar derivatives: evidence for the formation of serinol phosphate in vitro and in cultured cells

As mentioned in the introduction, any transamination reaction requires two substrates, one of which (the amino group acceptor) must contain a carbonyl function. Carbohydrates and phosphorylated carbohydrates, which contain by definition an aldehyde or keto functionality, are regularly present in the cytosol, where their metabolism takes place. We wondered therefore if these compounds (particularly the non-cyclic sugars such as glyceraldehyde or erythrose) could be also used as alternative amino group acceptors by some cytosolic transaminases.

**Fig. 4:**
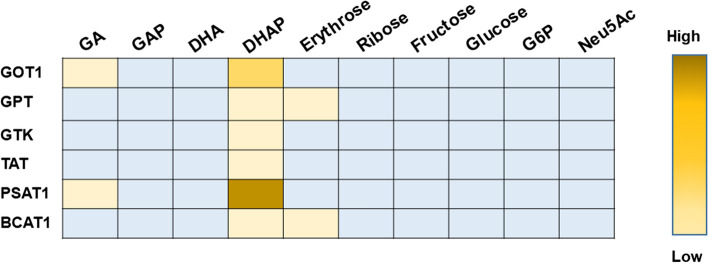
Assessment of the activity of the cytosolic transaminases towards several potential amino group acceptors that occur in the cytosol. The figure is based on the end-point spectrophotometric assays, in which the formation of the keto acid co-products’ pyruvate or α-KG was monitored using LDHA or GDH, respectively (see “Materials and methods” section). The extent of the transamination for each enzyme/amino acid combination is indicated semi-quantitatively by the intensity of the color. Light blue cells correspond to cases in which activity was essentially undetectable

To evaluate the possible transfer of the amino group from either L-Glu or L-Ala to alternative acceptors, we developed an assay based on the detection of the expected product keto acids (α-KG or pyruvate). These keto acids were detected using the appropriate dehydrogenases (GDH or LDHA, respectively), as described in the Materials and methods. The alternative amino group acceptors tested were: D,L-glyceraldehyde (GA), D,L-glyceraldehyde-3-phosphate (GAP), dihydroxyacetone (DHA), dihydroxyacetone phosphate (DHAP), erythrose, D-ribose, D-fructose, D-glucose, D-glucose 6-phosphate (G6P) and N-acetyl neuraminate (sialic acid; Neu5Ac). An overview of the results from this assay is presented in Fig. 4.

**Fig. 5:**
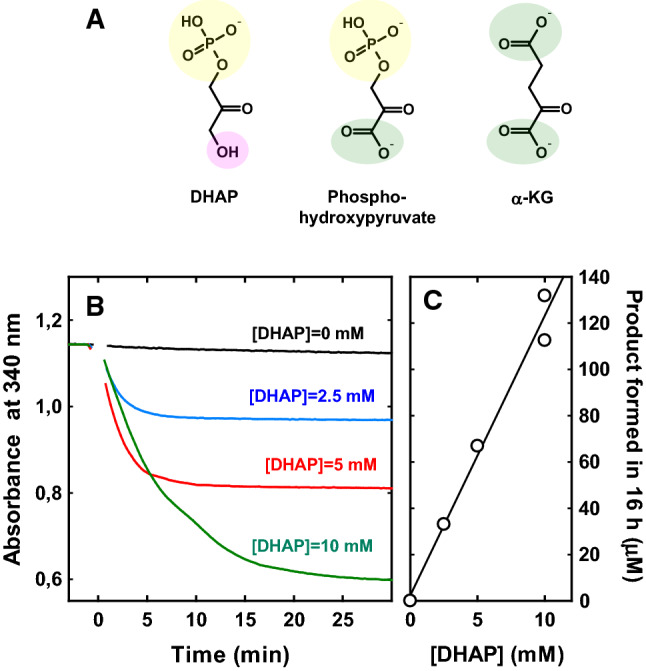
Reaction of PSAT1 with the glycolytic metabolite DHAP. **A** Comparison of the structure of DHAP with those of the two keto substrates preferred by PSAT1. **B** Readout of the assay for DHAP transamination. Reactions were carried out for 16 h at 37 °C, in 20 mM Hepes buffer (pH 7.2), also containing 0.5 mM DTT. The concentration of the amino group donor, L-Glu, was 1 mM; and the concentration of enzyme was 5 μM. At the end of the incubation, treatment with GDH (in presence of NADH and ammonium chloride) yielded the kinetic traces in the figure. **C** The endpoints of these traces (and hence the total amount of NADH consumed by GDH) reflected the amount of α-KG formed by the transaminase. This amount increased near-linearly with the concentration of DHAP. Based on these data, one can roughly estimate the catalytic efficiency of DHAP transamination by PSAT1 at around 0.04 M^−1^ s^−1^

The only carbonyl compound whose transamination could be detected consistently and unambiguously was DHAP, which reacted primarily with PSAT1 but also, to a lower extent, with GOT1, GPT and other transaminases. Possibly, this reactivity pattern was related to the structural similarity between DHAP and the two amino group acceptors preferred by PSAT1, namely phosphohydroxypyruvate and α-KG (Fig. 5A). Note that α-KG is also the preferred co-substrate for most of the six transaminases examined in this study (except GTK; Supplementary Table 1).

While the reaction of PSAT1 with DHAP was extremely slow, the amount of product formed increased linearly with the amount of DHAP in the reaction (Fig. 5B and C). The expected product of DHAP transamination is serinol phosphate [43–45], and indeed, a compound with the mass of serinol phosphate was observed by LC–MS to accumulate in the reaction mixture (Supplementary Fig. 6).

Could the slow transamination of DHAP observed in vitro be also occurring in vivo? To our knowledge, the occurrence of serinol phosphate in human tissues or cell lines has never been reported. To assess the presence of such a compound in cells, we analyzed metabolite extracts from HEK293T cells by LC–MS. Indeed, we observed a peak with the same *m*/*z* and elution time as a synthetic standard of serinol phosphate (Supplementary Fig. 7).

The observed peak was too weak for further analysis in MS2. While this low abundance prevented an additional confirmation of the identity of the compound, it was consistent with the remarkably low rate at which DHAP is transaminated in vitro. Nevertheless, our data did not rule out that the compound could be formed by routes not involving the transamination of DHAP.

## Discussion

There is no doubt that substrate specificity is a trait of enzyme behavior that (like other key properties such as catalytic efficiency and thermodynamic stability) has been largely shaped by natural selection [3, 4, 9]. Intuitively, natural selection must have refined most consistently the enzymes’ selectivity against ‘alternative’ substrates that are always present in the cell—such as intermediates in central metabolic pathways. The degree to which transamination reactions with specific metabolites have been suppressed over the course of evolution must be somehow correlated with the biological burden associated with the reaction themselves. While certain secondary activities may be tolerable for the cell or organism, reactions with other alternative substrates may be wasteful or otherwise damaging, which is expected to elicit a stronger negative selection towards reaction with these substrates [9].

Drawing inferences on the selective pressures that have shaped the specificity of a single enzyme from a single organism may be hazardous. Better hints have been obtained by examining the specificities of orthologous enzymes in different organisms [46]. Here, we argue that examining the situation of multiple enzymes of the same class in the same organism can offer interesting insights.

In fact, we have provided a broad (if still necessarily incomplete) and near-quantitative picture of the side activities of the human cytosolic transaminases—a group of enzymes that share the same type of catalyzed reaction and the same subcellular compartment. To interpret this picture, we will first spell out the factors that underlie substrate discrimination in general, and then, we will examine the cases of individual side reactions to put them in the context of human metabolism.

### The chemical and biological underpinnings of substrate discrimination

Schematically, the effective discrimination against an alternative substrate depends on the combination of two factors—physical–chemical constraints and natural (biological) selection. With ‘physical–chemical constraints’, we mean here that there are inherent limits to the ability of an active site to bind a good substrate while keeping out an alternative one. Ultimately, these limits are mostly due to the finite differences in binding energies associated with the structural differences between ligands [9].

Related to this, it might be expected that excluding from the active site compounds that are structurally very distinct from the preferred substrate should be particularly easy. For example, an enzyme that uses L-Asp as the preferred substrate should be inherently better at excluding from the active site the much bulkier and more hydrophobic L-Trp than the isosteric L-Asn. However, this is not always observed in practice: for example, GOT1 (which uses L-Asp as a preferred substrate) reacted with L-Trp about 80-fold faster than with L-Asn (Fig. 2 and Supplementary Table 5). Even more strikingly, MDH1 reduced IndPy (the keto acid derived from L-Trp) with remarkable effectiveness (Supplementary Fig. 4), whereas reactivity towards α-KSM (the keto acid derived from L-Asn) was essentially undetectable and hence presumably orders of magnitude slower. These counterintuitive observations can be explained in part by considering that while chemistry certainly sets the stage for selectivity, the actual degree of discrimination depends on biology, particularly on the evolutionary pressure for an enzyme to avoid the reaction with a given alternative substrate.

Indeed, as mentioned in the “Introduction” section, reactions with certain alternative substrates may be just biologically inconsequential (‘promiscuous’, according to the strict definition put forward by evolutionary biologists [5, 47]), whereas others could be frankly detrimental or else somewhat advantageous. As a consequence, discrimination against alternative substrates may follow different evolutionary dynamics, and be either subject to random drift or to some degree of selective (positive or negative) pressure.

**Fig. 6:**
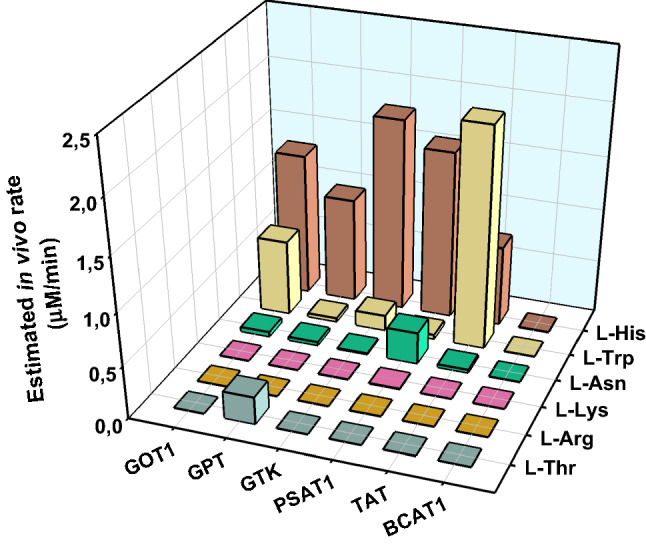
Estimated rates of transamination in the liver cytosol, for the six amino acids considered in this study. The in vivo rate of transamination for each enzyme–substrate combination was estimated as the product of three factors: the approximate concentration of the enzyme (last column of Supplementary Table 1; [48]), the average intracellular concentration of the substrate (second column of Supplementary Table 3; [49]) and the catalytic constants for the various enzyme–substrate couples (Supplementary Table 5)

To untangle the issue, it seems useful to estimate the actual extent at which a given side transamination proceeds in vivo (because intuitively reactions that are abundant in vivo are less likely to be deleterious) and also evaluate what can be the biological burden associated to such a reaction. Figure 6 summarizes the estimated rates of transamination (for each of the 36-amino-acid-enzyme couples considered in this paper) in the cytosol of hepatocytes. These estimated rates have been derived based on the parameters in Supplementary Tables 1, 3 and 5. In the following sections, the emerging patterns will be analyzed and discussed.

### Discrimination against L-Thr and L-Arg: favored by chemistry and justified by biology

Cytosolic transaminases, as a group, react extremely poorly with L-Thr and L-Arg, showing catalytic efficiencies always < 1 M^−1^ s^−1^ (Fig. 2). This pattern may be facilitated by chemical factors (since these two amino acids are very different from the preferred substrates used by the transaminases), but seems also biologically meaningful, since the keto acids derived from these two amino acids do not have any known metabolic role and may be difficult to reconvert into useful metabolites.

This is true in particular for L-Thr, as to our knowledge, no transaminase has ever been described in any organism that is specific for this amino acid. Transamination of L-Thr produces 3-hydroxy-2-ketobutyrate (Supplementary Table 2), which might conceivably cause some metabolic interferences, since it is rather similar to standard metabolic intermediates such as hydroxypyruvate and α-ketobutyrate. 3-hydroxy-2-ketobutyrate does not appear to have ever been detected in human tissues, according to the Human Metabolome Database (HMDB; [50]); however, its reduced counterpart 4-deoxythreonate (which could be produced, for example, by LDHA) is detected in urine and blood (e.g., Ref. [51]). Elevated concentrations of this compound have been observed in patients with diabetes mellitus Type I, and it was shown in a canine model of the disease that this compound is formed from L-Thr [40]. Our data suggest that, in vivo, 4-deoxythreonate can derive from an initial L-Thr transamination, operated particularly by the abundant liver enzyme GPT (Fig. 6). However, other routes (for example, involving the oxidative deamination of L-Thr) cannot be ruled out.

As for L-Arg, transamination of this amino acid is not considered part of its catabolism in humans [52, 53]. Nevertheless, it is assumed that L-Arg transamination can occur in vivo, since the reaction product, 2-keto-5-guanidino valerate (KetoArg, Supplementary Table 1), shows up at relatively high levels in patients with argininemia. Argininemia is an autosomal recessive inborn error of metabolism caused by a defect in the final step in the urea cycle, the hydrolysis of L-Arg to urea and ornithine (arginase deficiency). Accumulation of L-Arg metabolites, especially KetoArg, may trigger the central nervous system damage associated with argininemia [54]. The accumulation of KetoArg in argininemia suggests that this keto acid is a dead-end product and that there may be no ‘repair’ enzyme able to efficiently reconvert KetoArg into useful metabolites. The very limited propensity of the six cytosolic aminotransferases to act on L-Arg could be justified at least in part by a selective pressure to prevent the wasteful (and presumably toxic) formation of KetoArg.

### Interpreting the general discrimination of cytosolic transaminases against L-Lys and L-Asn

In contrast to the cases just described, the products of the transamination of L-Lys and L-Asn are not dead-end metabolic intermediates. Despite this, the transaminases tested in this work strongly discriminate against these two potential substrates for reasons that are not immediately obvious.

In particular, let us consider L-Lys. While this amino acid is structurally very different from the preferred substrates of the six transaminases, from a metabolic viewpoint, there seems to be no obvious need to avert its transamination. In fact, even though in humans, L-Lys is most commonly degraded through the so-called saccharopine pathway (which does not require transamination), formation of the corresponding α-keto acid, piperideine-2-carboxylate, is postulated as the first step of the pipecolic acid pathway—a minor degradative route which is reportedly predominant in the adult brain [30, 55].

However, the pipecolic acid pathway is relatively poorly characterized at the molecular level. In particular, it is not clear whether piperideine-2-carboxylate may be generated by transamination rather than, for example, oxidative deamination [55]. Related to this issue, our results indicate that none of the six cytosolic aminotransferases tested is a candidate to participating in this pathway. Another transaminase that has been suggested to act on L-Lys is GTL (glutamine transaminase L, which reportedly localizes both in the cytosol and in the mitochondria) [30, 56]. However, when we tested the activity of recombinant mouse GTL towards L-Lys, this enzyme performed very poorly, comparably to the human GTK (data not shown). Collectively, our data suggest that piperideine-2-carboxylate may not be formed at all by transamination, at least in the cytosol.

As for L-Asn, data from rat liver indicate that the routes of degradation are strongly compartmentalized. In the mitochondria, L-Asn is degraded largely via transamination (to produce α-KSM), followed by the ω-amidase reaction; whereas, the cytosolic catabolism of L-Asn proceeds mostly via the asparaginase reaction, to yield aspartate and ammonia [57]. In broad agreement with such a compartmentalization, our data indicate a general bias of cytosolic transaminases against this amino acid (Fig. 6). In the case of GOT1, the remarkably low activity towards L-Asn has been structurally explained by invoking the loss of electrostatic interactions and of proper positioning of this substrate in the enzyme active site [39], but it is difficult to envisage that the discrimination shown by all the tested transaminases arises from coincidentally similar structural/chemical constraints.

In terms of metabolic efficiency, it must be considered that while α-KSM, the Asn transamination product, can be reconverted to oxaloacetate by ω-amidase (Nit2) [58, 59], the process is energetically costly. Perhaps, more importantly, α-KSM is also relatively unstable: it is known to readily dimerize, and the dimer can undergo subsequent spontaneous reactions, forming various aromatic compounds [60]. It has been suggested that in vivo this compound could react with other α-keto acids or other reactive biomolecules [61]. All of this may justify selective pressures on the transaminases to discriminate against L-Asn. The conundrum however remains over why the formation of α-KSM is allowed and favored in the mitochondria [57], but apparently very repressed in the cytosol.

### L-His transamination: a tolerated metabolic mistake?

L-His is structurally quite different from all the ‘preferred’ substrates of the human cytosolic transaminases, yet it is measurably processed by all of them (Figs. 2 and 6).

From a biological viewpoint, L-His transamination could be the initial step in some ‘backroad’ degradation route for the amino acid. In fact, while L-His is generally catabolized via direct deamination by histidinase (histidine ammonia lyase) to form urocanate [62], a second pathway was proposed to occur in rat liver, beginning with some enzyme that transaminates L-His forming ImPy [63]. Although this compound is puzzlingly absent from the HMDB database, its detection in urine is well documented, particularly in cases of histidinemia, which is a relatively frequent, benign inborn error of metabolism associated with a deficiency of histidinase [64].

Which enzyme(s) produces ImPy? A cytosolic form of His transaminase has been partially purified and characterized from rat liver by Emes and Hassall [65]. The high activity of the rat enzyme against aromatic amino acids (Tyr, Phe) and its inability to use α-KG as an amino group acceptor [65] are strongly reminiscent of the properties of human GTK. This is in agreement with the fact that GTK is by far the most efficient enzyme in the transamination of L-His (Fig. 2). However, in the human liver (as opposed to rat liver), GTK is reportedly expressed at very low levels—much less than other transaminases such as GOT1 and GPT. As a result, while the latter enzymes transaminate L-His with low intrinsic efficiency, their contribution to L-His transamination in the human liver may be comparable to that of GTK (Fig. 6).

The picture which emerges from Fig. 6 is intriguing and may be explained by two distinct evolutionary scenarios. In a first scenario, considering that the formation of ImPy does not seem to have obvious pathological consequences and that it can be countered at least in part by the activity of GTK (Fig. 3B), the transamination of L-His can be considered biologically irrelevant, so that there is no selective pressure to prevent this reaction by other enzymes. In a second scenario, transamination of L-His may even be beneficial to some extent (e.g., as an overflow mechanism to avoid excessive accumulation of this amino acid) and hence favored by selection. In this scenario, the low-level transamination of L-His may be the result of collective catalysis (with most of the different transaminases contributing similarly to the process, at least in the human hepatocytes—Fig. 6).

The reaction of ImPy with a dehydrogenase produces imidazole-lactate [63]. It is believed that such a reduction is catalyzed by lactate dehydrogenase, although we found that the rate of reduction of ImPy by LDHA is extremely low compared with the rate with the enzyme’s normal substrate, pyruvate (Supplementary Table 4). Unlike ImPy, imidazole-lactate is present in the HMDB database and has been detected in the human urine, with a reported average excretion of 5.6 μmol/mmol creatinine [66].

### L-Trp transamination: striking a balance between degradation and recycling

L-Trp is an essential amino acid which, in addition to participating to protein synthesis, is a precursor of compounds such as serotonin and nicotinamide, and a potent immunomodulator. Therefore, in spite of irregular dietary supply, L-Trp homeostasis must be maintained quite strictly [67], implying a balancing between degradation and salvage. In animals, the main route for L-Trp catabolism is the kynurenine pathway, initiated by the enzyme tryptophan 2,3-dioxygenase [68, 69]. However, transamination of L-Trp is known to occur in mammals and is considered part of a secondary catabolic pathway, estimated to contribute to less than 3% to L-Trp degradation [69]. The product of L-Trp transamination, IndPy, has been detected in substantial amounts in patients suffering some rare forms of hypertryptophanemia [70]. In the HMDB database, IndPy is signaled as a microbial metabolite, as today it is well appreciated that significant amounts of this keto acid are produced by the human microbiota [71, 72].

Much earlier, however, tryptophan aminotransferase activity was demonstrated in rat liver, and ultimately, it was shown by Stanley et al. that most of this activity was attributable to tyrosine aminotransferase [40]. We substantially confirm this picture (Fig. 6) and suggest that another significant contribution to L-Trp transamination in vivo may come from GOT1 (Fig. 6). We also report here that GTK can perform the reaction with a respectable apparent efficiency, but using a different amino group acceptor and with a much lower K_M_ (Figs. 2 and 3A). However, the readily saturable activity of GTK on L-Trp seems to be offset by the much higher efficiency with which this transaminase generates L-Trp from its deaminated form, IndPy (Fig. 3). Accordingly, GTK in vivo may predominantly serve to catalyze the reconversion of IndPy into precious L-Trp, rather than the opposite reaction. This point is further expounded in the next section.

### A reappraisal of GTK as a multifunctional keto acid recycling enzyme

GTK stands out from all other aminotransferases in Supplementary Table 1 in several ways. First and foremost, it is very poorly active in the presence of α-KG as the amino group acceptor (and, conversely, of L-Glu as the amino group donor); instead, it uses preferentially KetoMet and phenylpyruvate as acceptors and L-Gln as a donor [42]. The reaction with L-Gln is substantially irreversible not only because this amino acid is particularly abundant in cells (Supplementary Table 3) and because the transamination product, α-ketoglutaramate (α-KGM) cyclizes spontaneously to a large extent [73], but, above all, due to the coupled reaction of ω-amidase, which consumes α-KGM [42, 58, 59]. Indeed, GTK is primarily involved in the salvage of amino acids [42].

Accordingly, the high activity of recombinant GTK with L-Trp and L-His may simply reflect the involvement of the transaminase in reactions that regenerate these amino acids from the respective keto acids [74]. The data presented particularly in Fig. 3B suggest that GTK, in cooperation with ω-amidase, can efficiently recycle IndPy and ImPy. In vivo, the combined action of these two enzymes may contribute to redress the mistaken transamination of L-Trp and L-His by other cytosolic transaminases. In a sense, this kind of reaction can be seen as a metabolite repair process, in which the useless keto versions of valuable amino acids are recovered in an energy-expensive fashion (see Ref. [74] and references therein).

**Fig. 7:**
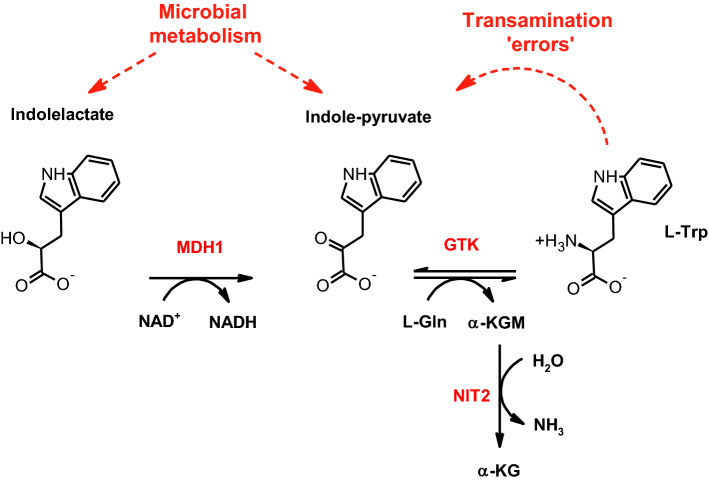
Proposed role of glutamine transaminase K (GTK; also known as KYAT1) in the regeneration of L-Trp from IndPy and indolelactate. The reaction of GTK with IndPy to produce L-Trp (see Fig. 3B) is rendered essentially irreversible by the action of ω-amidase (NIT2). We propose that this pathway may be integrated by the reduction of indolelactate to IndPy, operated by the cytosolic malate dehydrogenase (MDH1)

Related to the recovery of L-Trp, we have observed that MDH1 can quite efficiently reduce IndPy (Supplementary Fig. 4). In the cytosol, however, the greater abundance of NAD^+^ with respect to NADH would usually favor the opposite reaction, i.e*.*, the oxidation of indolelactate to produce IndPy. Based on this consideration, we suggest that in vivo MDH1 (through its side activity) and GTK could cooperate in the reconversion of indolelactate into L-Trp (Fig. 7). Once more, the process would be rendered essentially irreversible by the action of ω-amidase, which hydrolyzes α-ketoglutaramate (α-KGM), the co-product of the GTK reaction (Fig. 7). In principle, the conversion of indolelactate into IndPy could be also catalyzed by the peroxisomal 2-hydroxyacid oxidase 2 (also called glycolate oxidase) [75].

### Cytosolic transaminases are virtually inert against sugars and sugar phosphates.

Overall, the lack of reactivity of most of the cytosolic transaminases towards the carbohydrates tested seems to reflect the fundamental structural differences between keto acids and sugars, in particular (a) the lack, in sugars, of a carboxylate group adjacent to the carbonyl function and (b) the tendency of longer sugars (i.e., pentoses and hexoses) to exist mostly in cyclic form. The second factor may be prominent, since N-acetylneuraminate (which *does* contain a carboxylate adjacent to the keto group) did not appreciably react. All these observations, however, do not exclude some sort of selective pressure to avoid interferences between the amino acids and carbohydrate metabolism.

In keeping with the notion that compartmentalization is important in directing the metabolism [76], we speculate that evolution of eukaryotes might have led to a pool of cytosolic transaminases biased against using aldehydes as substrates, because aldehydes are often present in the cytosol as normal metabolic intermediates (for example, GAP or erythrose 4-phosphate). This is in contrast with the strictly mitochondrial localization of 4-aminobutyrate aminotransferase/GABAT, ornithine 5-aminotransferase/OAT and 5-aminolevulinate aminotransferase/AGXT2. These transaminases belong to a distinct structural subgroup of enzymes (labeled in Pfam ‘Aminotran_3’ family—PF00202) that typically prefer amino group acceptors whose carbonyl group is not adjacent to a carboxylate (in particular, aldehydes) [77]. In principle, therefore, these enzymes could accept more easily other aldehydes and ketones in their active sites. For example, ornithine aminotransferase reacted significantly more efficiently with glyceraldehyde 3-phosphate (Supplementary Fig. 8) and DHAP (not shown) than PSAT1. One evolutionary factor that might have favored the compartmentalization of these enzymes in the mitochondria is the relative paucity of aldehydes in the mitochondrial metabolic pathways, which limits the possibility of unwanted transaminations.

## Conclusions

The substrate specificity of enzymes is inherently imperfect, and minor reactions with alternative substrates occur continuously within cells. However, the extent and significance of this phenomenon has rarely been explored at the level of enzyme classes, as opposed to individual enzymes. As suggested in the Introduction, transaminases seem excellent candidates for beginning a broader exploration. Due to their ping–pong reaction mechanism, the active sites of transaminases must be able to accommodate at different times during the reaction at least two distinct (preferred) substrates—a feature which may facilitate the binding of other, alternative substrates.

Herein, we systematically characterized the reactivities of human cytosolic transaminases towards alternative (but physiologically available) amino group donors and acceptors. Our results provide valuable novel information on the cytosolic metabolism of L-Lys, L-His and (most strikingly) L-Trp. Furthermore, they suggest the plausible origin of some components of the human metabolome and imply that other unusual compounds that the cytosolic transaminases form in vitro (such as 3-hydroxy-2-ketobutyrate and serinol phosphate), despite having never been observed before, must be also formed in vivo, where they may become part of the so-called underground metabolism [78].

Last but not least, our results provide a better appreciation of the actual degree of specificity that these transaminases, as a group, have reached during evolution. Our data show some clear patterns of discrimination, which in most cases seem to have a plausible biological justification. They also suggest that evolution might have led to a cytosolic compartmentalization of only those transaminases most biased against reacting with simple (non-cyclic) sugars and sugar derivatives, which occur in the cytosol as normal metabolic intermediates.

### Supplementary Information

Below is the link to the electronic supplementary material.

Supplementary file1 (DOCX 2898 KB)

## Data Availability

The data presented in this study are available on request from the authors.

## Abbreviations

GOT1: Human glutamic-oxaloacetic transaminase 1
PSAT1: Human phosphoserine aminotransferase
GPT: Human alanine transaminase
GTK: Human glutamine transaminase K (also known as kynurenine aminotransferase 1—KYAT1)
BCAT1: Human branched-chain amino acid transaminase 1
TAT: Human tyrosine transaminase
MDH1: Human malate dehydrogenase (cytosolic)
LDHA: Human lactate dehydrogenase A
yNit3: Yeast ω-amidase
ADH: Alcohol dehydrogenase
GDH: Glutamate dehydrogenase
BSA: Bovine serum albumin
PLP: Pyridoxal 5′-phosphate
DHAP: Dihydroxyacetone phosphate
GAP: Glyceraldehyde 3-phosphate
α-KG: α-Ketoglutarate
α-KGM: α-Ketoglutaramate
α-KSM: α-Ketosuccinamate
ImPy: Imidazolepyruvate
IndPy: Indolepyruvate
KetoMet: 2-Keto-4-methylthiobutyrate
KetoArg: 2-Keto-5-guanidino valerate
HEPES: 4-(2-Hydroxyethyl)-1-piperazine ethanesulfonic acid
HMDB: Human metabolome database
