# Supplementary material for: Human cytosolic transaminases: side activities and patterns of discrimination towards physiologically available alternative substrates
Source: Cell Mol Life Sci. 2022 Jul 14;79(8):421. doi: 10.1007/s00018-022-04439-3 (PMC9283133; doi:10.1007/s00018-022-04439-3)
Supplement: Supplementary file 1 — Supplementary file1 (DOCX 2898 KB) [file 18_2022_4439_MOESM1_ESM.docx]

**Supplementary material for:**

**Human cytosolic transaminases: side activities and patterns of discrimination towards physiologically available alternative substrates**

Francesco Caligiore^a,b^, Erika Zangelmi^b^, Carola Vetro^b^, Takfarinas Kentache^a^, Joseph P. Dewulf ^a^, Maria Veiga-da-Cunha ^a^, Emile Van Schaftingen^a^, Guido Bommer ^a^, Alessio Peracchi^a,b^*

^a^ De Duve Institute and WELBIO, UCLouvain, Avenue Hippocrate 75, 1200 Brussels, Belgium

^b^ Department of Chemistry, Life Sciences and Environmental Sustainability,

University of Parma, 43124 Parma, Italy.

**Supplementary methods**

**LC-MS separation of the products arising from side transamination reactions**

Transamination reactions were conducted, quenched and deproteinized as described in the main text. The samples were injected on a 1290 HPLC system coupled to an ESI-QTOF iFunnel 6550 series MS (Agilent technologies) and analyzed through two distinct protocols, depending on the nature of the side chain (either basic or neutral) of the amino acid being transaminated.

**Basic amino acids -** The products arising from the transamination reactions of L-Lys, L-Arg and L-His were separated on a Kinetex F5 2.6 µm 2.1 mm × 150 mm column (Phenomenex, Maarsen, The Netherlands) through a procedure loosely based on the protocol described by Bonte and coworkers [1].

Mobile phases were A: 100% water, 0.05% formic acid, and B: 100% acetonitrile and 0.1% formic acid. The injection volume for all separations was 5 µL. Chromatographic elution was achieved under gradient conditions with a ﬂow rate of 0.35 mL/min.

Elution started with an isocratic step of 2 min at 0% B, followed by a linear gradient from 0% to 25% B (2– 4 min), 25% to 35% B (4– 8 min), and 35% to 95% B (8– 10.5 min). These conditions were maintained for 4.5 min before returning to 0% B in 0.2 min and equilibration at start conditions for 4.8 min. The total runtime was 20 min. The mass spectrometer was operated in positive mode using an electrospray ionization source under the following conditions: ESI spray voltage 3500 V, sheath gas 350°C at 11 L/min, nebulizer pressure 45 psig and drying gas 200 °C at 14 L/min. A range of m/z from 69 to 1150 was scanned.

**Neutral amino acids.** An LC-MS analysis of the products arising from the transamination reactions of L-Thr, L-Asn and L-Trp was performed on an ODS-4 column (150 x,2.1 mm; GL Biosciences) at a constant flow rate of 0.2 mL/min with mobile phase A, consisting of 5 mM hexylamine (Sigma-Aldrich, USA) adjusted to pH 6,3 using acetic acid (Biosolve BV); and phase B, consisting 90% Methanol in Water, 10 mM Ammonium formate adjusted to pH 8.5 with ammonia (Merck, Darmstadt, Germany). The injection volume was 5 μL.

The mobile phase profile consisted of the following steps and linear gradients: 0 – 2 min at 0% B; 2 – 6 min from 0 to 20% B; 6 – 17 min from 20 to 31% B; 17 – 36 min from 31 to 60% B; 36 – 41 min from 60 to 100% B; 41 – 51 min at 100% B; 51 – 53 min from 100 to 0% B; 53 – 60 min at 0% B. (from Coulier et al. [29]). The mass spectrometer was operated in negative mode using an electrospray ionization source under the following conditions: ESI spray voltage 3500 V, sheath gas 350°C at 11 L/min, nebulizer pressure 35 psig and drying gas 200 °C at 14 L/min. A range of m/z 70 to 1200 was scanned by combining 8122 transients leading to a 1 s cycle time. Extracted-ion chromatograms of the [M-H]-forms were integrated using Mass Hunter software (Agilent, CA, USA).

**Coupled spectrophotometric assays for individual transamination reactions**

When possible, the transamination reactions catalyzed by the human cytosolic aminotransferases were monitored spectrophotometrically. In most cases, this entailed using one or two coupling enzymes (the exception was L-His transamination, which could be monitored directly, by exploiting the peculiar absorption of ImPy; see main text). The coupled spectrophotometric assays were invariably conducted in phosphate buffer (pH 8.0), 37°C. Details for the individual assays are reported below.

**Threonine transamination assay -** The production of 3-hydroxy-2-ketobutyrate in L-Thr transamination reactions was quantitated through a discontinuous (end-point) assay, in which recombinant human lactate dehydrogenase (LDHA) was used to reduce the keto acid, with the concomitant oxidation of NADH.

Reactions were assembled in plastic tubes and the reaction mixture contained 20 mM sodium phosphate buffer (pH 8.0), 50 mM NaCl and 0.5 mM DTT, in addition to the amino group acceptor (either 0.5 mM α-KG or 3 mM pyruvate) and L-Thr. The concentration of enzyme was typically 5 μM.

The reaction was prepared in a final volume of 200 μl and incubated for 16 hours at 37 °C. In parallel, controls were performed in which either the enzyme or the keto acid was omitted. Then, 195 μl were taken from each of the reaction tubes and transferred to quartz microcuvettes; NADH was added to a final concentration of 0.2 mM and monitoring of the absorbance at 340 nm was started; after a couple of minutes about 3U of recombinant LDHA were added and the decrease in absorbance was followed until it stabilized. The overall drop in the concentration of NADH (ε_340_=6220 M^-1^s^-1^) was finally used to estimate the amount of α-keto acid formed during the 16-hours incubation.

**Asparagine transamination assay -** The transamination of L-Asn, to generate α-ketosuccinamate (α-KSM), was monitored through a continuous coupled assay with ω-amidase and MDH1. In these assays, ω-amidase (the enzyme used was the product of the *nit3* gene from *S. cerevisiae* [2]) converted α-KSM to oxaloacetate, which was then reduced to L-malate by MDH. As ω-amidase did have some marginal activity towards L-Asn (yielding L-aspartate, which in turn could react with the transaminase), concentration of the amidase was kept low compared to the transaminases and controls in which ω-amidase was added only at the end of the kinetics served to take into account this contribution to the observed rate.

The assay was performed at 37°C in 1-cm plastic cuvettes. The reaction mixture (1 mL final) contained 50 mM sodium phosphate buffer (pH 8.0) and 50 mM NaCl. MDH1 was 0.4 μM and yNit3 (ω-amidase) was 20 nM. L-Asn ranged between 0.5 and 30 mM, while the concentration of amino group acceptor was 0.5 mM (α-KG) or 3 mM (pyruvate).

**Tryptophan transamination assay -** The formation of Indolepyruvate (IndPy) in L-Trp transamination reactions was monitored spectrophotometrically through a continuous coupled assay with MDH1, which reduced quite efficiently this keto acid (see Results and Supplementary Figure 4) with the concomitant oxidation of NADH.

The assay was performed in 1-cm plastic cuvettes, equilibrated at 37°C in a thermostatted spectrophotometer. The reaction mixture (1 mL final) contained 50 mM sodium phosphate (pH 8.0), 50 mM NaCl, 0.25 mM NADH and 0.8 μM MDH1. L-Trp varied between 0.5 and 8 mM while the amino group acceptor was 0.5 mM (α-KG) or 3 mM (pyruvate). The concentration of the transaminase was 0.2 to 2.5 μM (depending on the enzyme under assay).

**Assays for the GTK-catalyzed transamination of different keto acids -** The transamination reactions of GTK with different keto acids, using L-Gln as the amino group donor, were monitored spectrophotometrically through a continuous coupled assay with ω-amidase and GDH as the indicator enzymes. The GTK reaction generated α-ketoglutaramate (α-KGM); ω-amidase hydrolyzed this compound to α-KG, which was eventually converted to L-Glu by GDH.

The assay was performed in 1-cm plastic cuvettes, equilibrated at 37°C in a thermostatted spectrophotometer. The reaction mixture (1 mL final) contained 50 mM sodium phosphate buffer (pH 8.0), 50 mM NaCl, 10 mM ammonium chloride, 1 mM L-Gln and 0.25 mM NADH, in addition to GDH (8 U) and ω-amidase (0.08 μM). The concentrations of GTK was typically 0.32 μM. The keto acids (amino group acceptors) tested, like IndPy, were typically poorly soluble in water and their actual concentrations were calculated retrospectively based on the overall extent of NADH reduction.

**Supplementary tables and figures**

**Supplementary Table 1 - The human transaminases tested in this work and their textbook reactions**

| **Enzyme/ gene symbol ^a^** | **Common name** | **Preferred amino donor substrates** | **Preferred amino acceptor substrates** | **Approximate enzyme concentration in hepatocytes (μM) ^b^** |
| --- | --- | --- | --- | --- |
| GOT1 | Aspartate aminotransferase | L-Glu, L-Asp | α-KG, Oxaloacetate | 27.9 |
| GPT | Alanine aminotransferase | L-Glu, L-Ala | α-KG, Pyruvate | 9.0 |
| GTK (KYAT1)^c^ | Glutamine transaminase K | L-Gln, L-Met, L-Phe, L-kynurenine | phenylpyruvate, 2-keto-4-methylthiobutyrate (KetoMet) | 0.1 |
| PSAT1 | Phosphoserine aminotransferase | L-Glu, L-Phosphoserine | α-KG, phosphohydroxy pyruvate | 10.7 |
| TAT | Tyrosine aminotransferase | L-Glu, L-Tyr | α-KG, *p*-hydroxyphenyl pyruvate | 2.0 |
| BCAT1 | Branched-chain amino acids aminotransferase | L-Glu, L-Val, L-Ile, L-Leu | α-KG, 3-Methyl-2-oxobutanoate, 4-Methyl-2-oxovalerate, 3-Methyl-2-oxovalerate, | 0.05 |

^a^ Structurally speaking, and according to the Pfam classification [3], GOT1, GPT, GTK and TAT are part of the ‘Aminotran_1_2’ family (PF00155); PSAT1 is part of the related ‘Aminotran_5’ family (PF00266) whereas BCAT1 is part of the distinct ‘Aminotran_4’ family (PF01063).

^b^ Values refer to monomers (i.e., to active sites), even though the enzymes in this list are all known to occur as homodimers. To estimate the concentration of the transaminases in hepatocytes we first assumed the cytosolic concentration of GOT1 to be 27.9 μM. This value stems from the following calculations. The number of GOT1 molecules per cell was taken as 19,187,233 (average of three measurements reported by Wisniewski et al. [4]) whereas the cytosolic volume was taken as 1.14x10^-12^ L (50% of the average volume for human hepatocytes, as reported by Yoshikado et al. [5]). The concentration of GOT1 (in moles/L) was then calculated by dividing the number of molecules by Avogadro’s number (N_A_) and by the cytosolic volume.

$$\left[ GOT1 \right]=\frac{n. molecules/cell}{N_{A}\times cytosolic volume}$$

Finally, the concentration of the other enzymes was calculated based on their relative (average) abundance with respect to GOT1. Relative abundances were taken from the PaxDB database (https://pax-db.org/) [6].

^c^Glutamine transaminase K is a multifunctional enzyme known by many names. The official gene symbol is KYAT1 (kynurenine aminotransferase 1) because the enzyme also acts towards the non-proteinogenic amino acid kynurenine. Furthermore, the enzyme also shows some β-lyase activity towards S-conjugated cysteine derivatives (a side activity common to many transaminases [7]) and is hence occasionally indicated as CCBL1 (cysteine conjugate β-lyase 1) [8]. Note also that KYAT1/GTK, contrary to the other enzymes in this list, is not strictly a cytosolic enzyme. A mitochondrial-associated form also exists. Apparently, alternative splicing the GTK mRNA, leading to the presence or absence, in the translated protein, of a mitochondrial leader sequence, directs the enzyme to either the cytosol or the mitochondria [9]

**Supplementary Table 2 - The six amino acids considered in this work as alternative substrates for cytosolic transaminases and their transamination products**

| **Amino acid** | **Expected transamination product(s)** |
| --- | --- |
|  |  |
|  |  |
|  |  |
|  |  |
|  |  |
|  |  |

**Supplementary table 3 – Reported intracellular concentrations of the six amino acids considered in this work and of some other relevant metabolites**

|  | **Intracellular concentration (mM)** | | | | |
| --- | --- | --- | --- | --- | --- |
| **Amino Acids (human)** | Liver | Liver |  | Skeletal muscle | Skeletal muscle |
| L-Thr | 0.64 ^a^ | 0.90 ^b^ |  | 1.03 ^c^ | 0.69 ^b^ |
| L-Lys | 0.29 ^a^ | 0.65 ^b^ |  | 1.15 ^c^ | 1.35 ^b^ |
| L-Arg | 0.036 ^a^ | - |  | 0.51 ^c^ | 0.65 ^b^ |
| L-Asn | 0.39 ^a^ | - |  | 0.47 ^c^ | - |
| L-Trp | 0.076 ^a^ | - |  | - | - |
| L-His | 0.88 ^a^ | 1.15 ^b^ |  | 0.37 ^c^ | 0.37 ^b^ |
|  |  |  |  |  |  |
| L-Glu | 5.3 ^a^ | 8.2 ^b^ |  | 4.38 ^c^ | 3.54 ^b^ |
| L-Ala | 3.7 ^a^ | 6.0 ^b^ |  | 2.34 ^c^ | 2.94 ^b^ |
| L-Gln | 6.0 ^a^ | 5.3 ^b^ |  | 19.50 ^c^ | 19.00 ^b^ |
|  |  |  |  |  |  |
| **Carbonyl compounds (rat)** |  |  |  |  |  |
| α-KG | 0.202 ^d^ | 0.120 ^e^ |  | 4.7 ^d^ | - |
| Pyruvate | 0.187 ^d^ | 0.258 ^f^ |  | 0.057 ^d^ | 0.095 ^f^ |
|  |  |  |  |  |  |
| DHAP | 0.045 ^d^ | 0. 043 ^f^ |  | 0.043 ^d^ | 0.017 ^f^ |

^a^ The amino acids concentrations in this column were estimated from the data reported by Barle et al. [10] assuming a ratio of 2.5 μL cell water per mg of protein.

^b^ Amino acid concentrations taken from Roth et al. [11].

^c^ Amino acid concentrations in skeletal muscle taken from Bergstrom et al. [12].

^d^ These concentrations in rat liver and muscle are taken from Albe et al. [13].

^e^ The cytosolic concentration of α-KG is from Soboll et al. [14].

^f^ Concentrations are taken from Veech et al. [15].

**Supplementary Table 4 – Activity of the human lactate dehydrogenase A (LDHA) towards different ketoacids**

| **Substrate** | **% activity** |
| --- | --- |
| Pyruvate | 100 |
|  |  |
| Oxaloacetate | 5.6 ^a^ |
| α-KG | 0.23 |
| α-ketobutyrate | 4.0 |
| Hydroxypyruvate | 2.5 |
| Mercaptopyruvate | 2.6 |
| Glyoxylate | 1.6 |
| Phenylpyruvate | 0.26 |
| Hydroxyphenylpyruvate | 0.24 |
| IndPy | 0.015 |
| ImPy | ~0.02 |
| α-KSM | <0.1^b^ |

The activity of recombinant LDHA was assayed in 50 mM phosphate buffer, pH 8.0, in the presence of 0.4 mM ketoacid and ~0.25 mM NADH.

^a^To measure the activity of LDHA with oxaloacetate, the ketoacid stock was prepared freshly; however, it is known that oxaloacetate can spontaneously decarboxylate to yield pyruvate, so this phenomenon can contribute to the observed rate.

^b^The reduction of α-ketosuccinamate (α-KSM) by a lactate dehydrogenase (presumably from bovine heart) was reported by Meister in 1953 [16]. While we confirmed that α-KSM is a substrate for LDHA, the reaction was too inefficient to be exploited in a continuous coupled assay for L-Asn transamination (data not shown).

**Supplementary Table 5 - Apparent catalytic parameters for the reactions carried out by cytosolic transaminases against six alternative amino acid substrates.**

|  | **0.5 mM α-KG** | | **3 mM Pyruvate** | |
| --- | --- | --- | --- | --- |
|  | *k_cat_/K_M_* (M^-1^s^-1^) | *K_M_* (mM) | *k_cat_/K_M_* (M^-1^s^-1^) | *K_M_* (mM) |
| **L-Thr** |  |  |  |  |
| GPT | 0.74 ± 0.20 | 2.8 ± 1.1 |  |  |
| BCAT1 | 0.17 ± 0.06 | >5 | - | - |
| **L-Arg** |  |  |  |  |
| GTK | - | - | 1.2 ± 0.1 | >10 |
| PSAT1 | 0.036 ± 0.008 | >10 | - | - |
| **L-Lys** |  |  |  |  |
| GTK | - | - | 0.9 ± 0.1 | >10 |
| PSAT1 | 0.023 ± 0.004 | >10 | - | - |
| **L-Asn** |  |  |  |  |
| GOT1 | 0.07 ± 0.02 | >15 | - | - |
| GPT | 0.15 ± 0.04 | >15 | - | - |
| GTK | - | - | 3.2 ± 1.0 | 6 ± 2 |
| PSAT1 | 1.2 ± 0.21 | >15 | - | - |
| TAT | 0.6 ± 0.05 | >15 | - | - |
| BCAT1 | 0.6 ± 0.10 | >15 | - | - |
| **L-Trp** |  |  |  |  |
| GOT1 | 5.6 ± 0.3 | >10 | - | - |
| GPT | 0.8 ± 0.2 | >10 | - | - |
| GTK | - | - | 490 ± 30 | 0.14 ± 0.03 |
| PSAT1 | 0.8 ± 0.1 | >10 | - | - |
| TAT | 226 ± 15 | >10 | - | - |
| BCAT1 | (0.2 ± 0.1) | >10 | - | - |
| **L-His** |  |  |  |  |
| GOT1 | 0.91 ± 0.05 | >10 | - | - |
| GPT | 2.1 ± 0.3 | >10 | - | - |
| GTK | (7 ± 2) | (0.6 ± 0.1) | 545 ± 120 | 1.5 ± 0.3 |
| PSAT1 | 2.8 ± 0.2 | >10 | - | - |
| TAT | 7.1 ± 1.5 | >10 | - | - |
| BCAT1 | 0.7 ± 0.1 | >10 | - | - |

Values refer to monomers. Assays were performed as described in the Methods, in the presence 0.5 mM α-KG or (for GTK only) of 3 mM pyruvate. Since none of the transaminases has ever been reported to show cooperativity, apparent kinetic parameters were obtained by fitting the experimental data (activity *vs.* substrate concentration) to the Michaelis-Menten equation or to a variation of the same that directly yields *k_cat_/K_M_*.

$v=\frac{{e\times K}_{M} [E]\left[ S \right]}{K_{M}+\left[ S \right]}$

Where v is the initial rate of the reaction, [E] is the total enzyme concentration, [S] the initial substrate concentration and e represents *k_cat_/K_M_*. For many couples, substrate saturation was not approached even at the highest amino acid concentration tested, preventing determination of accurate individual values for *k_cat_* and *K_M_*. However *k_cat_/K_M_* corresponds to the slope of the initial part of the Michaelis-Menten hyperbola, which could be reliably estimated even in the absence of saturation. Parameters are provided $\pm$ standard error of the fitting. For those reactions that were measured repeatedly on different days, the calculated kinetic parameters differed by less than 25% between individual measurements.


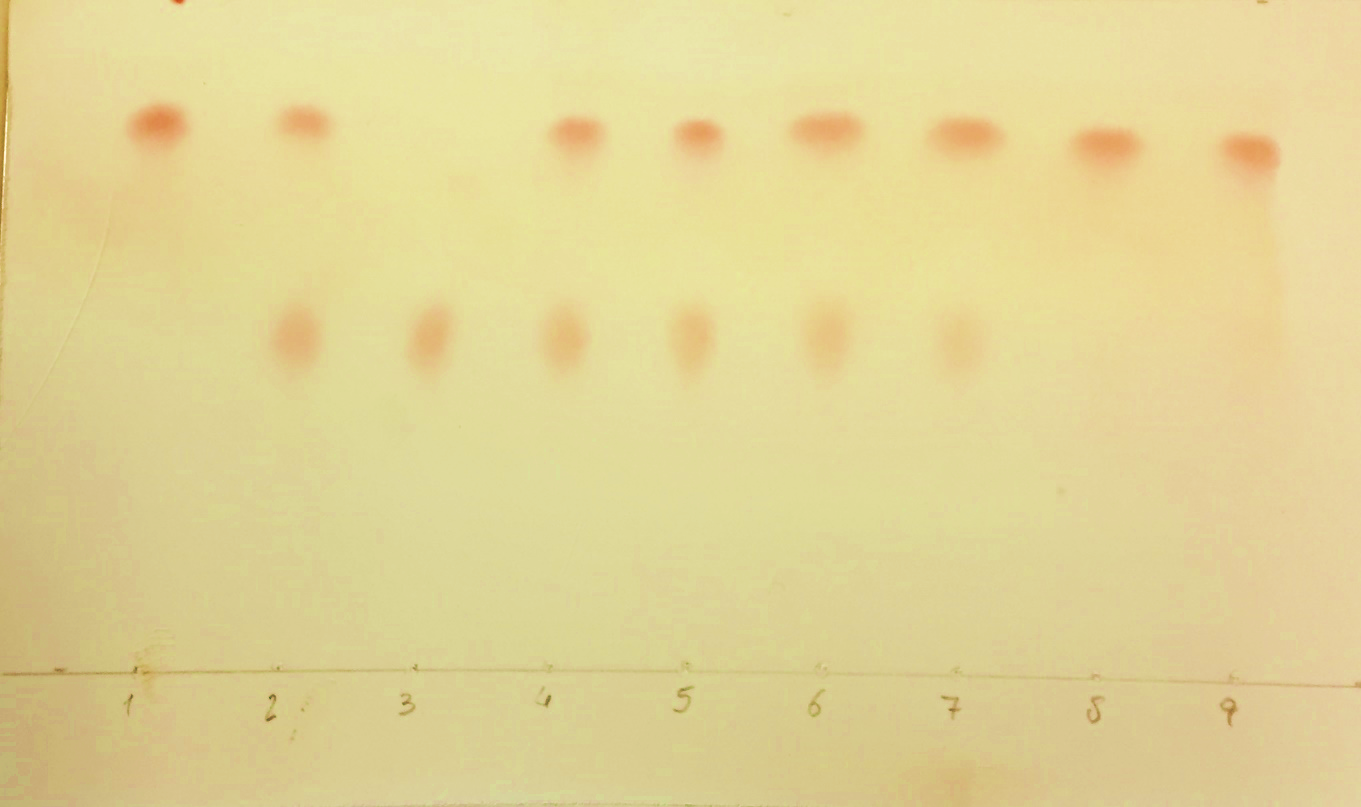


**W W+G G GOT1 GTK TAT PSAT1 BCAT1 GPT**

**Supplementary Figure 1 – Example of TLC assay, assessing the ability of the six cytosolic enzymes to catalyze the transamination reaction between L-Trp (5 mM) and glyoxylate (5 mM).** The reaction was carried out for 16 hours, as described in the Materials and Methods, and the expected amino acid product of this reaction was glycine. The first three lanes correspond to samples containing L-Trp, L-Trp + Gly and Gly without the presence of any enzyme, in order to show the substantial separation and coloring of the two amino acids (substrate and predicted product) on the plate.

**Supplementary Figure 2 – LC-MS chromatograms showing formation of the expected keto acid product for L-Thr transamination.** The reaction of BCAT1 (2.5 μM) with L-Thr (2.5 mM) and α-KG (1 mM) was carried out for 15 hours at 37°C in 20 mM Hepes pH 8.0. A peak with the mass corresponding to 3-hydroxy-2-ketobutyrate (monoisotopic mass 118.0266; mass of the deprotonated form, 117.01933) was detected in samples containing L-Thr, α-KG and the enzyme BCAT1 (bottom panel) but not in samples containing only α-KG and L-Thr (upper panel ) or α-KG and BCAT1 (not shown). In the same reaction, L-Glu was also appearing very clearly.

**Supplementary Figure 3 – Formation of the expected keto acid products for the transamination of L-Lys, L-Arg and L-His, carried out by GTK.** Samples in which the three reactions had occurred were analyzed by LC-MS in positive mode, as described in the Materials and Methods. In all cases, a peak with the mass corresponding to the expected product was observed in samples containing α-KG or pyruvate and the enzyme **GTK** but not in samples in which the enzyme was missing. (Masses for the various compounds are indicated in the panels and correspond to the monoprotonated species).

**Supplementary Figure 4 – Human MDH1 reduces with substantial efficiency the alternative substrate indolepyruvate (IndPy).** (A) Dependence of the rate of oxaloacetate reduction (mmoles of product per mmole of enzyme per second) on the initial concentration of oxaloacetate. Na-phosphate 50 mM, NaCl 50 mM, pH 8.0, 37°C. The concentration of NADH was ~220 μM whereas the concentration of MDH1 was 1.6 nM. (B) Time-courses of the reaction of MDH1 at different concentrations of indolepyruvate (due to the poor solubility of IndPy in water, its concentration was estimated based on the endpoints of the reactions themselves). Conditions: Na-Phosphate 50 mM, NaCl 50 mM, NADH ~220 μM, pH 8.0, 37°C. The enzyme (0.4 μM) was added after pre-incubating the reaction mixture at 37°C, for about 20 min, during which time the tautomerization of IndPy (from the enol form, predominant in the concentrated ethanolic stock, to the keto form predominant in aqueous solution [17]) occurs spontaneously and the absorbance at 340 nm stabilizes. (C) Dependence of the rate of IndPy reduction on the concentration of substrate. (D) Effect of increasing concentrations of *meso*-tartrate on the reduction rate of oxaloacetate (blue bars) and IndPy (red bars). Since *meso*-tartrate had to be added in rather high concentration to observe an appreciable effect, the experiments were conducted at pH 7.2 (where the buffering efficiency of sodium phosphate is greater than at pH 8.0) and the pH of the *meso*-tartrate stock was also adjusted to ~7.2. When measuring the reduction of oxaloacetate, the concentration of MDH1 was 1.6 nM and the concentration of oxaloacetate was 180 μM. For experiments with IndPy, the concentration of enzyme was 160 nM and the formal concentration of IndPy was 250 μM. (E) Effect of increasing concentrations of succinate on the reduction rate of oxaloacetate (blue bars) and IndPy (red bars). Experiments were conducted as those in panel D.

**Supplementary Figure 5 – UV-visible spectral properties of imidazolepyruvate (ImPy), the ketoacid derived from transamination of L-His.** The spectrum of ImPy (0.8 mM in phosphate buffer pH 8) is shown in red; the spectrum of an equimolar concentration of L-His in blue. Inset: dependence of the absorption at 386 nm on ImPy concentration. Linear fitting of the data indicates an extinction coefficient of 1,400 M^-1^ cm^-1^.

**Supplementary Figure 6 - Observation of a compound showing the mass of serinol phosphate upon prolonged incubation of PSAT1 (5 μM) with DHAP (5 mM) and L-Glu (2 mM).** The reaction was carried out in 20 mM Hepes pH 7.2 – 1 mM DTT, at 37°C for 48 hours. The reaction mixture was then extracted and analyzed by LC-MS as described in the Materials and Methods. (A) Structures of the compounds involved in the transamination reaction (substrates and products). Notably, in our ESI-LC-MS method, DHAP was mainly detectable as a dimer that forms in-source during the ionization process. (B) LC-MS chromatogram for a control reaction mixture in which the enzyme was absent. The presence of a small amount of α-KG in the sample reflected perhaps a contamination of the L-Glu stock. (C) LC-MS chromatogram of the reaction mixture that contained the enzyme.

**Supplementary Figure 7 – LC-MS evidence for the presence of serinol phosphate in extracts of cultured HEK293T cells.** (A-C) Extracted ion chromatograms of a species with m/z 170.0224 (expected for serinol phosphate in deprotonated form) in 3 different biological replicates of HEK293T cells. (D) A serinol phosphate standard (produced as described in the legend of Supplementary Figure 6) was added to the metabolite extract of HEK293T cells prior to LC MS injection. (E) A comparison of the chromatogram of the ion found in cells (median of the 3 replicates) with the standard shows perfectly coincident retention times.

**Supplementary Figure 8 –Activities of the cytosolic enzyme PSAT1 and of the mitochondrial ornithine aminotransferase (OAT; from mouse) towards D,L-glyceraldehyde-3-phosphate (GAP).** (A) Structures of GAP and of the amino group acceptors preferred by PSAT1 (phosphohydroxypyruvate and α-KG) and OAT (α-KG and glutamate semialdehyde). In each case, the reactive carbonyl group is highlighted. (B) Comparing the extent of GAP transamination by PSAT1 (5 μM) and mOAT (2.5 μM). Reactions were conducted for 16 h in 20 mM Hepes buffer (pH 7.2), also containing 0.5 mM DTT, at 37° C. The D,L-GAP concentration was 10 mM (5 mM of each isomer) and the concentration of the amino group donor (L-Glu) was 0.5 mM. At the end, the extent of transamination was assessed by adding sodium phosphate pH 8.0 (50 mM final concentration), NADH (~0.19 mM final concentration) and ammonium chloride (10 mM final); after incubating at 30°C for a few minutes, GDH (1U) was added and the decrease in absorbance at 340 nm could be related to the amount of α-KG formed.

**References**

1. Bonte R, Bongaerts M, Demirdas S, et al (2019) Untargeted metabolomics-based screening method for inborn errors of metabolism using semi-automatic sample preparation with an UHPLC-orbitrap-MS platform. Metabolites 9:1–18

2. Peracchi A, Veiga-Da-Cunha M, Kuhara T, et al (2017) Nit1 is a metabolite repair enzyme that hydrolyzes deaminated glutathione. Proc Natl Acad Sci U S A 114:E3233–E3242

3. Mistry J, Chuguransky S, Williams L, et al (2021) Pfam: The protein families database in 2021. Nucleic Acids Res 49:D412–D419

4. Wiśniewski JR, Hein MY, Cox J, Mann M (2014) A “proteomic ruler” for protein copy number and concentration estimation without spike-in standards. Mol Cell Proteomics 13:3497–3506

5. Yoshikado T, Toshimoto K, Nakada T, et al (2017) Comparison of methods for estimating unbound intracellular-to-medium concentration ratios in rat and human hepatocytes using statins. Drug Metab Dispos 45:779–789

6. Wang M, Weiss M, Simonovic M, et al (2012) PaxDb, a database of protein abundance averages across all three domains of life. Mol Cell Proteomics 11:492–500

7. Cooper AJL, Krasnikov BF, Niatsetskaya Z V., et al (2011) Cysteine S-conjugate β-lyases: Important roles in the metabolism of naturally occurring sulfur and selenium-containing compounds, xenobiotics and anticancer agents. Amino Acids 41:7–27

8. Cooper AJL (2004) The role of glutamine transaminase K (GTK) in sulfur and α-keto acid metabolism in the brain, and in the possible bioactivation of neurotoxicants. Neurochem Int 44:557–577

9. Malherbe P, Alberati-Giani D, Köhler C, Cesura AM (1995) Identification of a mitochondrial form of kynurenine aminotransferase/glutamine transaminase K from rat brain. FEBS Lett 367:141–144

10. Barle H, Ahlman B, Nyberg B, et al (1996) The concentrations of free amino acids in human liver tissue obtained during laparoscopic surgery. Clin Physiol 16:217–227

11. Roth E, Mühlbacher F, Karner J, et al (1987) Free amino acid levels in muscle and liver of a patient with glucagonoma syndrome. Metabolism 36:7–13

12. Bergstrom J, Furst P, Noree LO, Vinnars E (1974) Intracellular free amino acid concentration in human muscle tissue. J Appl Physiol 36:693–697

13. Albe KR, Butler MH, Wright BE (1990) Cellular concentrations of enzymes and their substrates. J Theor Biol 143:163–195

14. Soboll S, Horst C, Hummerich H, et al (1992) Mitochondrial metabolism in different thyroid states. Biochem J 281:171–173

15. Veech RL, Lawson JW, Cornell NW, Krebs HA (1979) Cytosolic phosphorylation potential. J Biol Chem 254:6538–6547

16. Meister A (1953) Preparation and enzymatic analogues of asparagine. J Biol Sci 200:571–589

17. Kaper JM, Gebhard O, van den Berg CJ, Veldstra H (1963) Studies on indolepyruvic acid. II. Ultraviolet Spectrophotometry. Arch Biochem Biophys 103:475–487
